# Supplementary material for: IRF8 deficiency causes anxiety-like behavior in a sex-dependent manner
Source: bioRxiv. 2025 Sep 4:2025.09.02.673764. Preprint. [Version 1] doi: 10.1101/2025.09.02.673764 (PMC12424652; doi:10.1101/2025.09.02.673764)
Supplement: Supplement 1 — Figure S1. A. Total distance (m) that mice traveled in the OFT arena. B. The number of mouse entries to the closed arm (left) and open arm (right) in EPM test. C. The number of mouse entries to the dark box (left) and light box (right) in LDB test. Bar graphs show mean and SEM; Orange (WT) and blue (IRF8KO) circles are individual data points. Asterisks indicate significant differences (*p.adjusted<0.05, **p.adjusted<0.01, ***p.adjusted<0.001). Figure S2. A. Venn diagrams displaying the shared genes between two types of DEGs. The top diagram compares upregulated DEGs (FDR<0.01) in male IRF8KO microglia with those in female IRF8KO microglia. The bottom diagram compares downregulated DEGs in male IRF8KO microglia with those in females. B. Heatmaps for mitochondrial Complex I (NAD(P)H+ dehydrogenase; left) and Complex IV (cytochrome c oxidase; right) genes expression in adult, female IRF8KO and WT microglia. C. Heatmaps for mitochondrial Complex I (NAD(P)H+ dehydrogenase; left) and Complex IV (cytochrome c oxidase; right) genes expression in P9 and adult female IRF8KO and WT microglia. The datasets deposited in GSE266424 was re-analyzed. D. Flow cytometry of male, adult microglia stained with a DCFDA dye. WT (magenta) and IRF8KO (orange) microglia were presented in the left. The mean fluorescent intensity (MFI) was analyzed in the right. E. Volcano plots presenting the expression of genes that are differentially expressed in females (pink) and males (blue) microglia from WT (left) and IRF8KO (right). Male microglia specifically expressed Y-linked genes, such as Kdm5d and Ddx3y, while X chromosome genes, such as Xist and Tsix, were expressed only in females. Note that there was a substantial number of genes irrelevant to sex chromosomes that were upregulated or downregulated in a genotype-specific manner. [file media-1.pptx]

## Slide 1
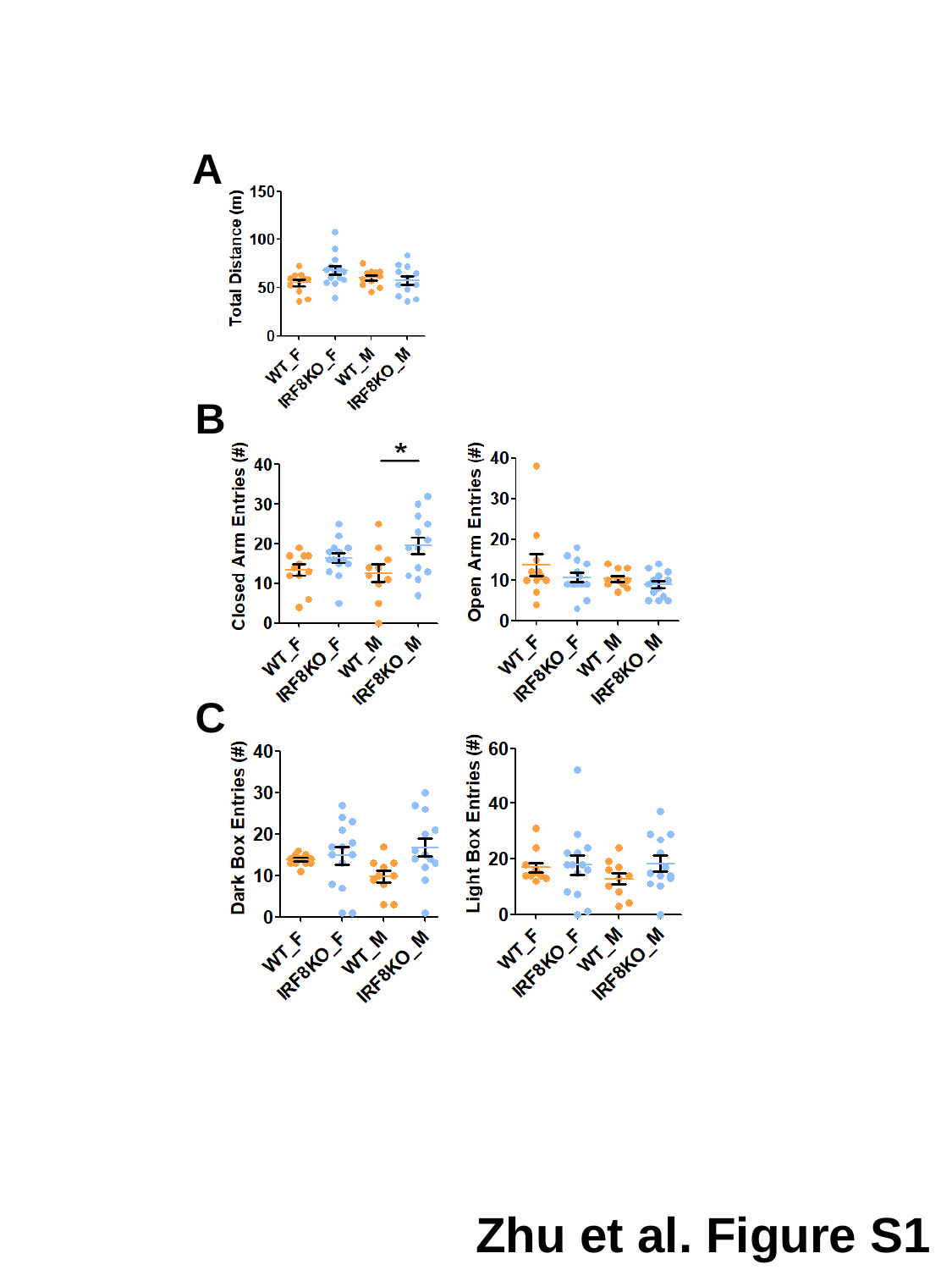

A
B
C
Zhu et al. Figure S1

## Slide 2
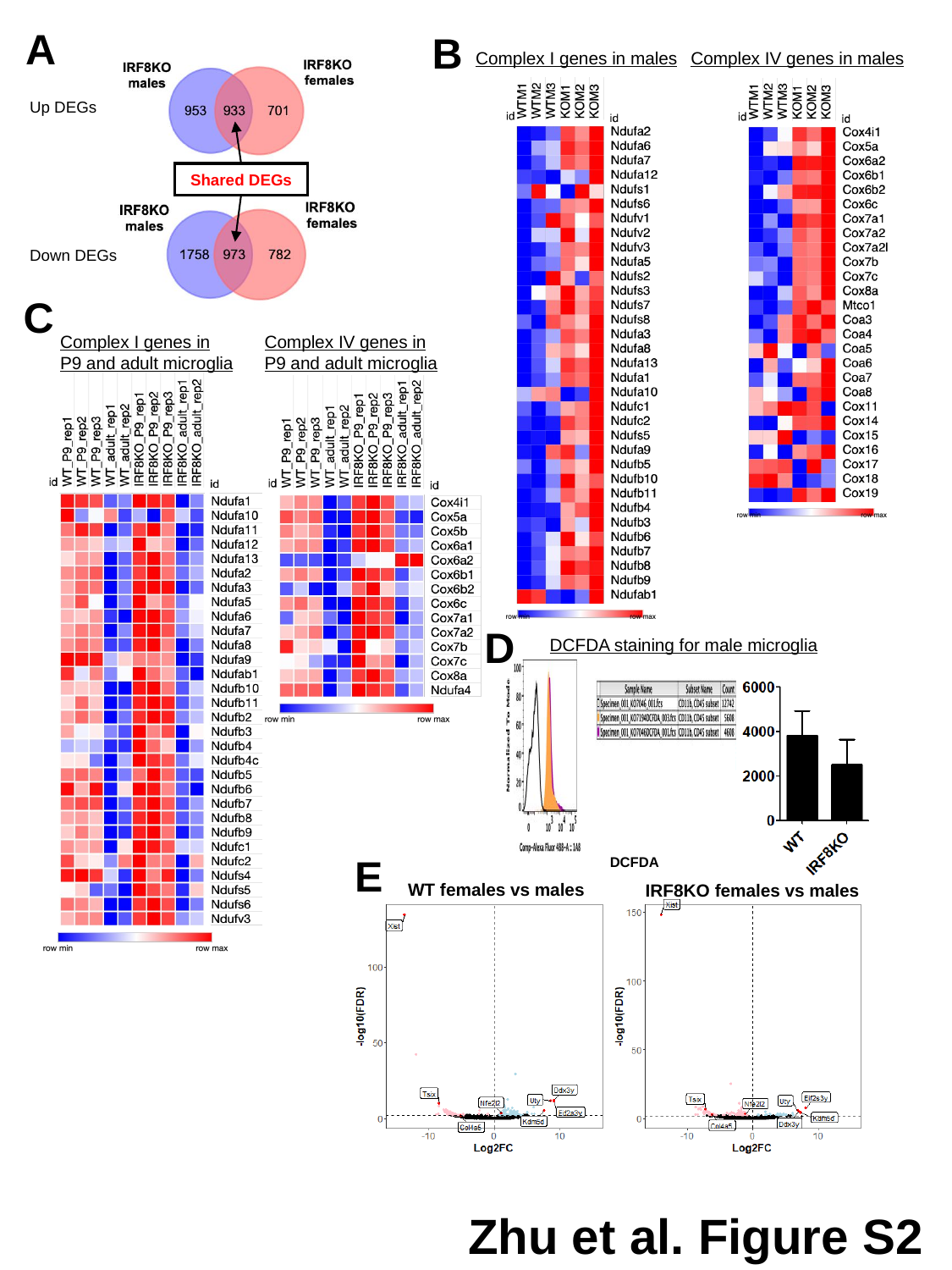

A
B
Complex I genes in males
Complex IV genes in males
Up DEGs
Shared DEGs
Down DEGs
C
Complex I genes in P9 and adult microglia
Complex IV genes in P9 and adult microglia
D
DCFDA staining for male microglia
E
DCFDA
WT females vs males
IRF8KO females vs males
Zhu et al. Figure S2
